# Heat Stroke-Induced Takotsubo Cardiomyopathy With a Concomitant Transient Brugada-Like Electrocardiogram

**DOI:** 10.7759/cureus.42605

**Published:** 2023-07-28

**Authors:** Osamu Sasaki, Toshihiko Nishioka, Hideki Sasaki

**Affiliations:** 1 Cardiology, Saitama Medical Center, Saitama Medical University, Kawagoe, JPN; 2 Internal Medicine, Mombetsu General Hospital, Mombetsu, JPN; 3 Cardiovascular Surgery, Nagoya City University East Medical Center, Nagoya, JPN

**Keywords:** electrocardiogram, coronary artery disease, takotsubo cardiomyopathy, brugada syndrome, heat stroke

## Abstract

A 79-year-old man presented with impaired consciousness, fever with a body temperature of 41.1°C, and an electrocardiogram showing significant ST segment elevation in leads V1-6, coved-type ST elevation in leads V1-3, and partial right bundle branch block. Echocardiography revealed notable left ventricle dysfunction with apex-based akinesis. Coronary angiography confirmed severe obstructive lesions but left ventriculography displayed the distinct apical ballooning of takotsubo cardiomyopathy (TC). This case highlights the presence of atypical electrocardiographic patterns in TC influenced by comorbidities, supporting the diagnosis of TC despite concurrent obstructive coronary artery disease.

## Introduction

Takotsubo cardiomyopathy (TC) is characterized by symptoms and electrocardiographic changes mimicking acute myocardial infarction (AMI), typically observed in older women and provoked by various causes such as emotional and physical stress [1-3]. Heat stroke is clinically characterized by a core body temperature surpassing the critical threshold of 40°C, and some reports have implicated this condition as a potential etiological factor in the development of TC [4, 5]. However, the presence of TC accompanied by an atypical electrocardiogram (ECG) has not been documented or definitively explained in this case. We present a case of heat stroke-induced TC accompanied by coronary artery disease, along with intriguing electrocardiographic observations.

## Case presentation

A 79-year-old man was transported to the emergency department complaining of a fever lasting two days and impaired consciousness. His medical background encompassed a variety of conditions, including a history of lung cancer, renal failure, diabetes, and abdominal aortic aneurysm, concurrent with regular hemodialysis. Upon presentation to the emergency room, the patient was in a somnolent state. His vital signs were: blood pressure of 122/74 mmHg; heart rate of 111 beats per minute; and body temperature of 41.1°C.

Laboratory findings revealed liver dysfunction, renal dysfunction, and a significant elevation of cardiac markers and catecholamines. His blood tests revealed that his aspartate aminotransferase level was 622 IU/L (8-38 IU/L); alanine transaminase was 399 IU/L (4-43 IU/L); lactate dehydrogenase was 1218 IU/L (115-280 IU/L); urea nitrogen was 52.4 mg/dL (8-20 mg/dL); creatinine was 6.68 mg/dL (0.40-1.10 mg/dL); phosphorus was 4.5 mg/dL (2.5-4.5 mg/dL); calcium was 8.7 mg/dL (8.6-10.2 mg/dL); sodium was 141 mEq/L (135-145 mEq/L); potassium was 4.9 mEq/L (3.5-5.9 mEq/L); parathyroid hormone was 167 pg/mL (10-65 pg/mL); creatine kinase was 312 IU/L (0-170 IU/L); cardiac troponin I was 14.1 ng/mL (0.00-0.09 ng/mL); adrenaline was 293 pg/mL (0-100 pg/mL); noradrenaline was 10,776 pg/mL (100-450 pg/mL); and dopamine was 533 pg/mL (0-20 pg/mL). Arterial blood gas analysis revealed hypoxemia (measured acid-base balance of the blood (pH): 7.383, partial pressure of oxygen (PaO_2_): 49.4 mmHg, partial pressure of carbon dioxide (PaCO_2_): 31.9 mmHg, serum bicarbonate (HCO3): 18.6 mEq/L; room air). The chest x-ray showed cardiomegaly (cardio-thoracic ratio, 61%) and pulmonary congestion.

**Figure 1:**
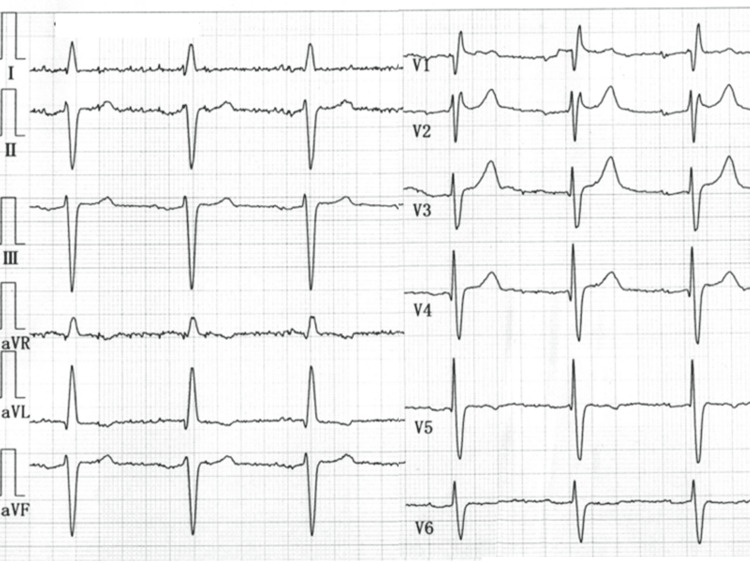
The patient's electrocardiogram taken six months prior to this admission

**Figure 2:**
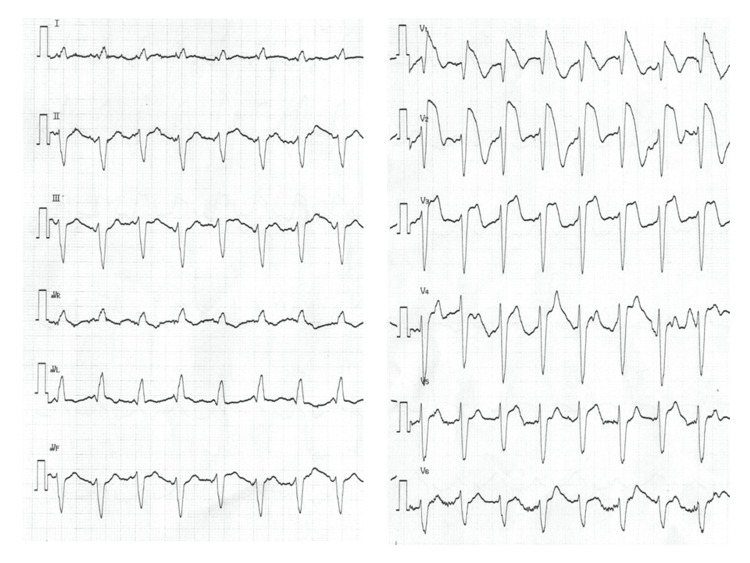
The patient's electrocardiogram taken with a body temperature of 41°C

In contrast to an electrocardiogram (ECG) taken six months prior to admission, which presented a slight ST elevation in leads V2-4 (Figure 1), the ECG obtained in the emergency room at the time the patient showed a body temperature of 41.1°C exhibited distinctive ST segment elevation across leads V1-6, notably demonstrating pronounced coved-type ST elevation in leads V1-3, accompanied by concurrent presentation of partial right bundle branch block (Figure 2).

**Figure 3:**
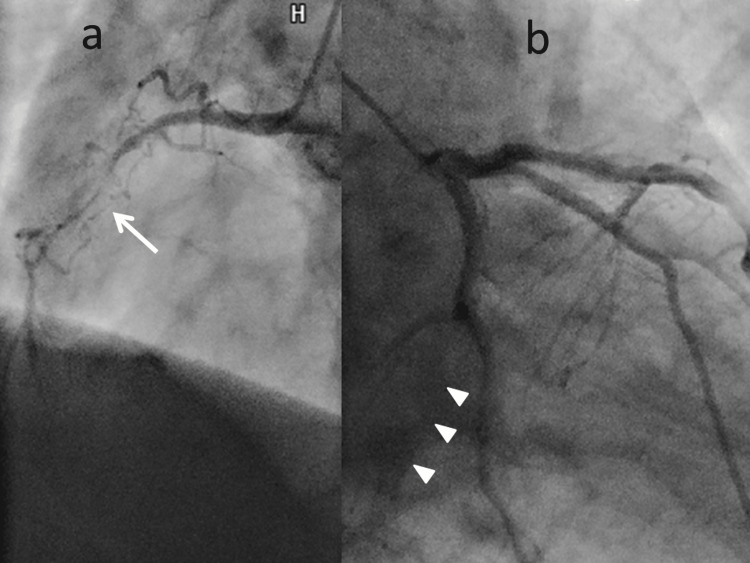
Coronary angiogram Coronary angiographic findings showcase concomitant subtotal and total obstructive lesions. a) A noteworthy coronary angiogram depicting 99% stenosis of the right coronary artery (white arrow); b) Complete occlusion of the left circumflex artery (arrowhead)

The patient remained without a history of faintness and did not have any reported cases of Brugada syndrome within their family. Transthoracic echocardiography (TTE) revealed impaired left ventricular function characterized by akinesis at the apex of the left ventricle (LV). Comparison with a previous TTE obtained six months prior to admission demonstrated preserved left ventricular contraction without any evidence of regional wall motion abnormalities (left ventricular ejection fraction: 50.8%). Following admission, coronary angiography (CAG) revealed an obstructive lesion within the hypoplastic right coronary artery (RCA), along with total occlusion of the left circumflex artery (LCx) (Figure 3).

**Figure 4:**
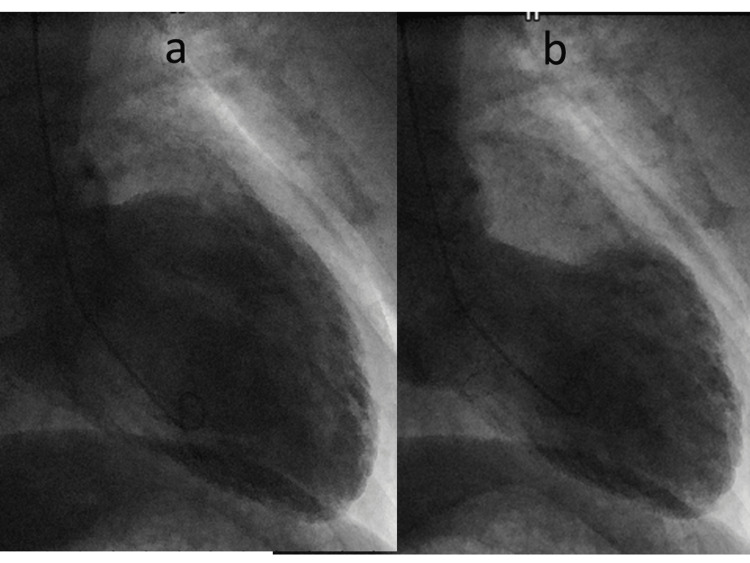
Left ventriculographic findings Left ventriculogram in the right anterior oblique view in diastole (a) and systole (b) isolated midventricular and apical ballooning are evident.

Collateral blood flow to both the RCA and LCx was facilitated by the left anterior descending artery. Left ventriculography exhibited diminished systolic function characterized by akinesis of the mid-ventricular and apical regions of the LV, while the basal segment exhibited preserved contraction (Figure 4).

**Figure 5:**
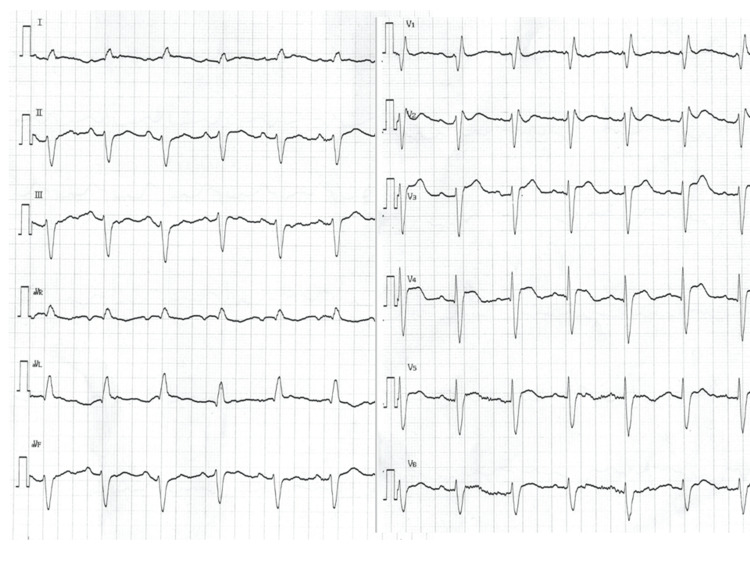
The patient's electrocardiogram taken with a body temperature of 37.2°C

A follow-up ECG after four hours revealed a notable trend toward ST-segment resolution. Particularly noteworthy was the marked descent of the ST elevation observed in leads V1-3, prominently shifting towards the baseline, with a body temperature of 37.2°C (Figure 5).

Despite the substantial presence of multiple coronary artery stenoses in this patient, a notable change in wall motion characterized by apical wall akinesis was evident, representing a distinct alteration compared to the wall motion observed prior to the current hospital admission. Subsequent TTE unveiled the amelioration of the left ventricle wall motion abnormality. Subsequently, the level of consciousness recovered as the fever decreased. However, respiratory failure, heart failure, and liver dysfunction demonstrated rapid progression. The patient and his family refused any invasive procedures, including mechanical ventilation. The condition of the patient progressively deteriorated, ultimately leading to a fatal outcome characterized by multiple-organ failure and septic shock on day three of hospitalization.

## Discussion

We identified two important clinical issues.

First, electrocardiographic findings from TC may exhibit variability, with certain cases displaying deviations from the characteristic ECG patterns documented previously. In general, the electrocardiographic findings in TC often resemble those observed in AMI, characterized by ST segment elevation on the ECG. A comparative analysis of electrocardiographic discrepancies between TC and AMI highlights a notable absence of ST segment elevation in lead V1 and ST depression in lead aVR as differentiating features in TC [6]. However, the ECG in our case reveals a notable and pronounced elevation of the ST segment specifically in leads V1-3; in particular, the presence of ST elevation in lead V1 without concurrent ST depression in lead aVR deviates from the typical electrocardiographic patterns seen in TC, rendering these observations atypical of TC.

Previous studies have demonstrated that fever can serve as an influential environmental factor in patients with Brugada syndrome, potentially modifying the condition [7-9]. The sodium voltage-gated channel alpha subunit 5 (SCN5A) mutation induces a significant loss of transmembrane current and subsequent ST-T changes. Another report highlighted the manifestation of heat-induced Brugada-type ECG findings in the absence of any discernible genetic abnormalities [10]. In the present patient, the ECG showed ST change in leads V1-6, with marked ST elevations in leads V1-3 with high body temperature. A discernible trend was subsequently observed as the ST elevation progressively shifted closer to baseline, with the marked ST elevations being transient in nature, particularly during episodes of elevated temperatures. While no genetic analyses were conducted, we speculate that the distinctive electrocardiographic changes observed in leads V1-3 in our patient align with characteristic findings associated with Brugada syndrome. The mechanisms underlying the electrocardiographic abnormalities observed in our patient could involve a fusion of the Brugada electrocardiographic pattern and the electrocardiographic manifestations associated with TC, both of which are likely influenced by elevated body temperatures [4, 5, 7-9]. However, the precise mechanisms underlying the atypical electrocardiogram observed in this case remain unclear. A comprehensive understanding requires further investigations and the accumulation of additional cases to shed light on this matter.

Second, the manifestations of TC despite the presence of coronary artery lesions suggest a potential etiology that is not solely reliant on the coronary artery anatomy. In the present case, CAG revealed multiple obstructive lesions within the RCA and LCx. Importantly, however, the observed wall motion abnormalities of the LV cannot be solely attributed to the coronary artery lesions. The Mayo criteria for TC show the absence of obstructive coronary artery disease or angiographic evidence of acute plaque rupture [3], so our case may not be deemed representative of TC based on the current diagnostic criteria. However, other recent reviews and reports indicate that patients with coronary artery disease may also develop TC [11-14]. Our patient also showed TC with significant coronary artery lesions, consistent with those reports. The presence of coronary artery disease thus may not represent the exclusion criteria for TC. We propose a prudent departure from utilizing the presence or absence of coronary artery lesions as a determinant. Instead, we advocate for an informed decision-making approach that assigns precedence to the comprehensive medical history, clinical presentation, laboratory findings, electrocardiography, imaging results, and environmental factors of the patient.

## Conclusions

In conclusion, electrocardiographic findings associated with TC in patients with concurrent comorbidities, including subclinical disease, may exhibit distinctive features, deviating from the electrocardiographic patterns typically observed in isolated cases of the condition. We propose that a clinical diagnosis of TC should be predicated upon the individualized profile of the patient, encompassing the underlying medical history, clinical presentation, various clinical data, and contextual environmental factors, regardless of the presence or absence of coronary artery lesions.
